# Spanish Validation of the PALMS (Physical Activity and Leisure Motivation Scale)

**DOI:** 10.3390/ijerph191610064

**Published:** 2022-08-15

**Authors:** Sagrario Pérez-de la Cruz, Juan Jose Gonzalez-Gerez, Óscar Arellano de León, Antonio Vargas Rodriguez

**Affiliations:** 1 Department of Nursing, Physiotherapy and Medicine, University of Almería, 04120 Almería, Spain; 2 Physical Education Department, Training and Improvement Division, National Police School, 05004 Ávila, Spain

**Keywords:** sport, exercise, motivation, reliability, validity, exploratory factor analysis

## Abstract

Although there is abundant evidence supporting an active lifestyle, it is necessary to promote the practice of physical activity among the population. To understand this phenomenon, several studies have been conducted to describe the reasons why people participate in sports activities. The PALMS (Physical Activity and Leisure Motivation Scale) was created as a comprehensive tool to measure the reasons for practicing sports. This tool consists of 40 items related to sports and is designed for the young and adult population. Each of the subscales is formed of five items (mastery, enjoyment, psychological condition, physical condition, appearance, the expectations of others, affiliation, competition/ego) that reflect the possible reasons for practicing sports. This study sought to validate the PALMS in the cultural context of Spain, for the type of population for which it is designed. In total, 596 voluntary participants completed the study from Spain, aged 18 to 53, who regularly practice sports. The adequacy of the model obtained in the exploratory study was confirmed, since a model composed of eight factors and 40 indicators in total was obtained. The parameters were statistically significant (*p* < 0.05) and the factor loadings presented values greater than 0.5. Regarding internal consistency, the values of Cronbach’s alpha and those of the composite reliability were above 0.8. In conclusion, the validation of the Spanish PALMS proved to be a valid and reliable measurement instrument for the evaluation of the reasons that lead the population to perform sports physical activity.

## 1. Introduction

Physical exercise is one of the fundamental pillars of health promotion. The World Health Organization (WHO) states that physical inactivity is currently the leading cause of mortality. Thus, a sedentary lifestyle is associated with a twofold increased risk of cardiovascular disease, diabetes, obesity, increased risk of colon cancer, hypertension, osteoporosis, lipid disorders, depression, and anxiety [1]. In contrast, regular exercise or physical activity has shown promising links for the prevention and treatment of many of these diseases [2,3]. Therefore, it is important to motivate people to become more physically active in their daily lives.

One of the factors that stimulates and maintains people’s participation in physical activity is motivation. Several types of motivation have been shown to influence effort during exercise sessions and intention to continue exercising [4]. Both intrinsic and extrinsic motives are found among the factors that motivate individuals. For example, individuals who are intrinsically motivated to participate in a physical activity (motivated by factors related to the activity, such as enjoyment or skill development and mastery) tend to participate for a longer period of time, since, in comparison with people with extrinsic motivation (they perform a physical activity due to factors that are not related to the activity itself, such as social, work, affective reasons, etc.), they obtain an improvement in health or good physical appearance as a reward [5]. Therefore, by determining people’s motivation for an activity, health professionals can use this knowledge to create awareness that will not only be beneficial at the individual level but will also help the community to reduce lifestyle-related diseases. More specifically, given this information, health professionals can develop effective interventions to motivate people to participate in physical activity, thereby increasing adherence to activity, promoting motivation at the individual level and at the community level, helping to reduce diseases caused by lack of activity.

During the last decades, a series of instruments have been designed to analyze the type of motivational regulation that a person has towards sports practice. One of such scales is the Sport Motivate Scale (SMS) [6], originally developed in French and validated in Spanish in successive studies [7,8]. This instrument consists of 28 items, which are divided into three subscales, constituting a total of seven factors to analyze an athlete’s self-determination. However, this instrument has presented certain limitations due to cultural, educational, or socioeconomic reasons. Subsequently, the authors of the SMS developed a second version (SMS-II), which performed better than the former version, proving to be reliable and valid, although the authors recognized that it should be further improved in future research [9].

Other measurement scales that can be used for the same purpose include the Exercise Motivation Scale (EMS) [10], the Exercise Motivation Inventory (EMI) [11], the Physical Activity Motivation Measure (MPAM) [12], the Physical Activity Motivation Measure-Revised (MPAM-R) [13] and the Perceived Successful Exercise Questionnaire (POSQ-E) [14]. The researchers who developed these instruments based their content on different approaches to the notion of motivation for physical activity, compiling a diverse number of reasons to justify why a person engages in physical activity.

Subsequently, and based on the qualitative data and comparing the results of the extensive version of the Participation Motivation Questionnaire (PMQ) (a scale used to determine motivation for sports practice, but with a high descriptive content that provided little information on the establishment of a theory of motivation) [15], we continued to work on obtaining a scale that gathers the greatest amount of information. Comparing the results of the 50-item version of the PMQ [16], the MPAM [12] and MPAM-R [13], a 73-item measure was developed with Likert scale responses (scored from 0 to 5 points). This new measure was called the Recreational Exercise Motivation Measure (REMM) [17]. This new measure consisted of assessing intrinsic (mastery and enjoyment), extrinsic (psychological fitness, physical fitness, and appearance), and extrinsic social factors (expectations, affiliation, and competence in relation to others/self).

As the implementation of this measure evolved, a shortened version of the REMM was developed, based on a combination of empirical and theoretical factors. First, Morris and Roger [18] determined the most appropriate structure and length for a shortened version of the REMM. Second, a statistical analysis was performed to validate this version. Finally, the five strongest categories were selected from the previous eight to create a 40-item measure, making the PALMS (Sport and Leisure Motivation Scale) equivalent to the REMM in relation to its robust psychometric characteristics. To corroborate this hypothesis, Chowdhury [19] administered the PALMS to 202 volunteers aged between 18 and 71 years who volunteered to play sport in various sporting institutions in Australia. The results of a confirmatory factor analysis indicated that the PALMS had a robust factor structure (CMIN/DF (chi-square/degrees of freedom) = 2.22; NFI (Normed Fit Index) = 0.95; CFI (comparative fit index) = 0.97; RMSEA (root mean square error of approximation) = 0.078). Zach et al. [20] translated the scale into Hebrew and validated it by applying the scale to a sample of 678 people, aged 9–89 years, who were regularly physically active. The results obtained showed good internal consistency for each of the subscales, which ranged from 0.63 to 0.96. More recently, Van Lankveld has validated this scale in Dutch, showing results similar to those obtained in previous validations [21].

Based on previous studies conducted in Australian and Israeli populations, the aim of this study was to carry out a cross-cultural adaptation of the PALMS in the Spanish language in a sample of people who practiced sports from different geographical locations in Spain. As a secondary objective, an initial psychometric analysis of the functioning of the instrument in this sample and its validation was carried out.

## 2. Materials and Methods

### 2.1. Participants

The sample consisted of 596 participants aged between 18 and 53 years (M = 35.8 years, SD = 11), practicing non-competitive physical activities (weight training, aerobic activities, aquatic activities, etc.), belonging to two institutions (National Police School (ENP) of Avila and Faculty of Health Sciences of the University of Almeria), both in Spain.

The sample consisted of 343 men (M = 41.2 years, SD = 8.7) and 253 women (M = 31.6 years, SD = 12.9). Their participation was voluntary. Information sheets were distributed to all participants. If they agreed to participate after reading the information sheet, completion of the questionnaires was considered to indicate consent.

A series of demographic variables were gathered, such as sex, age, marital status, level of studies, time spent practicing sports, perception of physical fitness and health status. All participants resided in Spain and had a perfect understanding of spoken and written Spanish.

### 2.2. Scale

The Physical Activity and Leisure Motivation Scale (PALMS) consists of 40 items. The volunteer was asked to answer statements, thinking about the reasons that motivated them to practice their physical activity/sport. The exploratory factor analysis revealed that 8 sub-scales emerged as expected, but the other expectations’ sub-scales were split into two: family/friends’ expectations and health/employers’ expectations (enjoyment, employers’ expectations, affiliation, competition/ego, psychological condition, appearance, physical condition, families’ and friends’ expectations, health professionals’ and employers’ expectations and mastery). They were asked to be as spontaneous as possible when responding to the questions and to avoid over-reflecting on each response. The answers were rated from 1 (strongly disagree) to 5 (strongly agree) [22]. The Spanish version of The Physical Activity and Leisure Motivation Scale can be found in Supplementary Materials.

### 2.3. Procedure

Ethical approval for this study was granted by the Bioethics Committee of the University of Almeria (Ref: UALBIO2020/022) after authorization by the director of the ENP, the director of the Department of Physical Education of the ENP, and the University of Almeria. The study was conducted between April and May 2021 and followed the guidelines of the International Test Commission (ITC) for adapting tests across cultures.

The instrument was administered during an in-person (M = 405, 67.95% of the total sample) and/or virtual interview (M = 191, 32.05% of the total sample). The interviewer took note of the answers given by the interviewee. All participants were informed of the purpose of the study, and of the confidentiality of responses and the data obtained. They were also informed that there were no right or wrong answers and were asked to respond with sincerity and honesty.

Following Hambleton’s guidelines [23], a back-translation strategy was adopted. The scale was translated into Spanish and, subsequently, another group translated the scale back into English and compared it with the original. This comparison revealed full coincidence. Subsequently, the battery of items was subjected to an evaluation by three experts in the subject who were external to the study. They considered that the items were pertinent to measure what was intended; in addition, they considered that the wording was appropriate.

Before applying the scale to the final sample, the complete battery was first administered to a sample of 50 participants, who confirmed their understanding of the battery of questions. This was the definitive scale that would be applied to the final study sample.

During the final administration of the scale, the participants were informed about the procedure for completing the questionnaire, which insisted that they leave no item unanswered, as well as the anonymity of their answers, with their participation being completely voluntary. The time required to complete the scale was approximately ten minutes.

### 2.4. Statistical Analysis

Qualitative variables were described by absolute (*n*) and relative (%) frequencies and the mean and standard deviation (SD) for quantitative variables.

To determine the suitability of the proposed items, as well as the factor structure of the questionnaire, an exploratory factor analysis was performed using the principal components method with Varimax rotation. After the model was generated in the exploratory study, a confirmatory analysis with structural equations was performed using the maximum likelihood extraction method.

Model fit was assessed using the following fit indices: Goodness of Fit Index (GFI), Adjusted Goodness of Fit Index (AGFI), Comparative Fit Index (CFI), Normed Fit Index (NFI), Tucker Lewis Index (TLI), and Root-Mean-Square Error of Approximation (RMSEA). Internal consistency (Cronbach’s alpha and composite reliability), convergent validity (factor loadings and average variance extracted = AVE), and discriminant validity (square root of the AVE) were also studied.

## 3. Results

Below is the table of the population participating in the validation study (Table 1).

### 3.1. Exploratory Factor Analysis (EFA)

The exploratory factor analysis was developed following the Principal Component methodology with Varimax rotation (Table 2). This analysis revealed the existence of eight factors with an eigenvalue greater than 1. The criterion for assigning an item to each factor was that its factor load was above 0.3. The eight factors explained 74.3% of the total variance.

The correlation matrix showed an outstanding number of correlations (89.1%), which obtained a value above 0.3, with a determinant equal to 4.84 × 10^−16^. The Bartlett’s sphericity test result determined the non-independence of the variables (Bartlett’s test = 8245.64 (l.g. = 780), *p* < 0.001). Additionally, the Kaiser–Meyer–Olkin test (KMO test), which seeks to show the adequacy of the sample, had a result of 0.937 and the communalities were higher than 0.55. Finally, the set of Measures of Sampling Adequacy (MSA) values were above 0.85. In summary, the above values give viability to the factor analysis of the correlation matrix.

### 3.2. Confirmatory Factor Analysis (CFA)

The confirmatory analysis was performed with structural equations using the maximum likelihood extraction method, after the model generated in the exploratory study.

**Figure 1:**
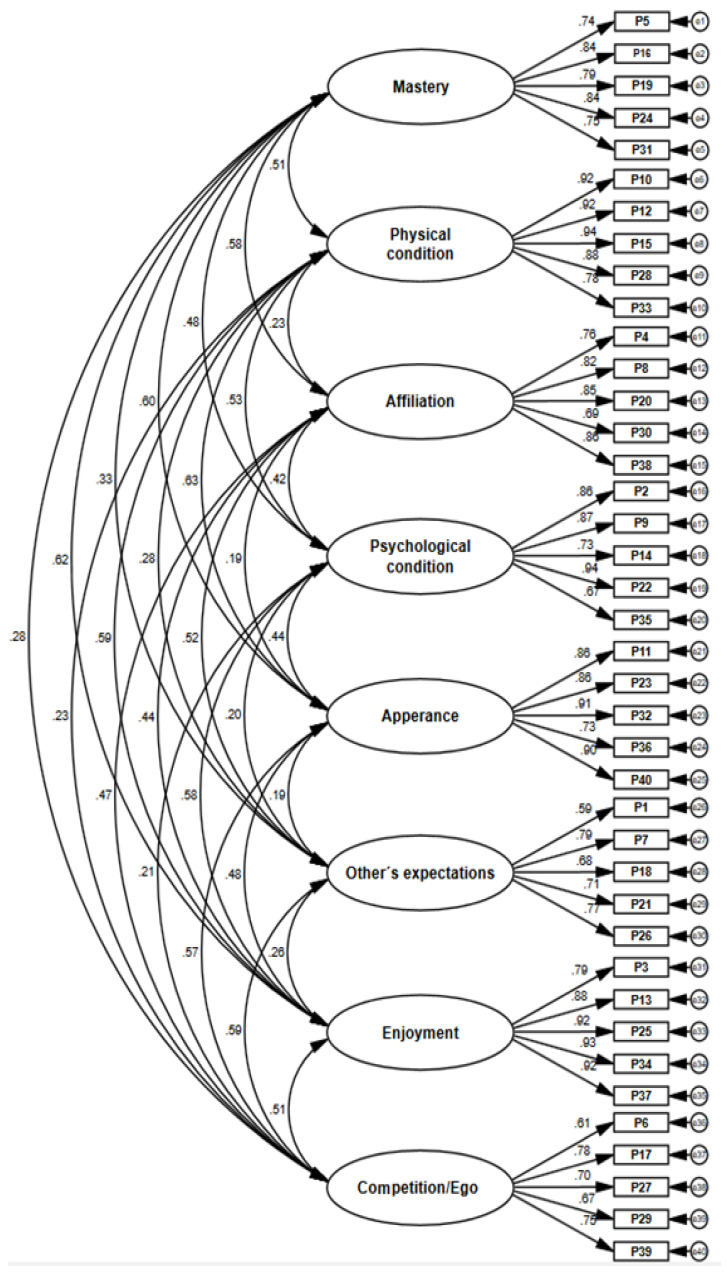
Confirmatory factor analysis.

The adequacy of the model obtained in the exploratory study was confirmed, since a model composed of eight factors and 40 indicators in total was obtained. The parameters were statistically significant (*p* < 0.05) and the factor loadings presented values higher than 0.5 (Figure 1). All the indicators saturate satisfactorily with each of their latent variables and the AVE values were higher than 0.60, which shows convergent validity. Regarding internal consistency, Cronbach’s alpha and composite reliability values were above 0.8 (see Table 3).

Regarding the model fit (Table 4), the various fit indices analyzed were adequate, indicating that the factorial structure of the proposed scale was appropriate.

### 3.3. Measurement Invariance

The results of the analysis of the measurement invariance between men and women are shown in Table 5.

A preliminary analysis in which the goodness of fit of the structure of the scale in the sample of both genders was examined separately, showed that both models were satisfactory, as indicated by the CFI and RMSEA goodness-of-fit indices of both models (Base men and female base), with all the estimated parameters being statistically significant (*p* < 0.05). The results of the invariance analysis reflected that the configurational invariance was satisfactory. The invariance of factor loadings (metric invariance) was also tested, finding a more restricted level of invariance than the previous one and also resulting in a good fit since the indices did not change significantly.

Next, the invariance of the intercept of the items (scalar invariance) was examined, obtaining satisfactory results. Thus, the results reflect that the RMSEA remained in acceptable magnitudes (<0.05) in addition to producing changes of less than 0.002 between the CFI of the models. Therefore, these results indicate that the measurement properties remain satisfactorily invariant in the tested invariance criteria (configurational, metric, and scalar) between men and women.

## 4. Discussion

The PALMS [19], which has presented solid psychometric characteristics, was the basis for this validation in Spanish. Compared with the 202 volunteers recruited by Chowdhury DR [19], this study involved 596 participants from different regions of Spain. Zach S [20], validated the PALMS in Hebrew, recruiting 678 participants, and concluded that it is a reliable scale for measuring motivation to engage in physical exercise. It should be noted that among the subjects who participated in Chowdhury’s study [19] there were athletes who participated in the Australian Football League, as opposed to those who participated in the present study and in Zach’s study [20], which included regular athletes who were not competing in any professional or semi-professional league. This confers greater applicability to this study because it is a more varied sample with diverse motivations. Thus, the data collected related to motivational aspects may apply to a greater proportion of the population.

To confirm the psychometric characteristics of the PALMS in the Spanish context, a confirmatory factor analysis with structural equations was performed using the maximum likelihood extraction method, obtaining the factors “improvement, physical condition, socialization, psychological condition, physical appearance, external expectations, fun, and competitiveness/ego”. The first factor with its five corresponding items deals with the improvement of the subject’s sports skills, together with the seventh factor, which is related to the feeling of happiness provided by the sports activity, and which is associated with intrinsic motivation. The second factor is directly related to the maintenance of an optimal state of health and its consequences. The third factor involves the relationship to and interaction with society, improving the sociability of the subject. The fourth factor is a factor in which motivation refers to the improvement in coping with situations of stress and pressure. The fifth factor is a factor related to extrinsic motivation. As discussed in the introduction to this article, the subject performs the activity due to factors that are not related to the physical activity itself. The same happens to the sixth factor, referring to external expectations. The last factor is associated with motivation to the competitive spirit in front of the group of the subject who performs the physical activity.

The internal consistency of the different factors was above 0.8; these values are similar to those obtained by Zach et al. [20], which were between 0.63 and 0.96, and to those of Chowdhury DR [19] with 0.79; therefore, the psychometric data of these studies are in agreement. Within this internal consistency, it is worth analyzing that the psychological condition factor is placed above the remaining factors, and therefore it is outstanding and motivating for the performance of physical activity. As for convergent validity, three factors (improvement, socialization, and external expectations) have lower AVE values than the remaining factors, making these the weakest factors.

As mentioned in the results, the factor loadings presented values above 0.5. The sum of the highest factor loadings belonged to the physical condition factor and the fun factor, whereas the sum of the items corresponding to the ego competitiveness factor and the external expectations factor yielded the lowest result of all those analyzed.

It can be affirmed that the results obtained in this validation with a Spanish population support the applicability of this questionnaire as a measure of a wide range of motives for participation in physical activity. Among the factors measured by the PALMS, these can be classified as aspects of intrinsic motivation (domain, enjoyment subscales) and extrinsic motivation (the other six subscales). This is based on both the results of the second-order factor analysis [24] and self-perception theory [25]. The motives can be classified into two groups: body/mind motives (psychological condition, physical condition, and appearance) and social motives (varied expectations, affinity, and competitiveness) [24]. Additionally, each of the eight motivational subscales have implications for intentions and behavior related to physical activity. For example, a high score on the appearance subscale might reflect an intention to engage in activities that improve appearance and body build, such as weight training for muscle development or yoga to increase flexibility. Similarly, a high-affinity score could lead people to join teams or clubs with group practice. The PALMS demonstrated an adequate factorial structure, initial validity, and reliability, but also great applicability to physical activity contexts. In addition, PALMS offers a comprehensive view. The analysis of the participants’ motives, which in previous or similar questionnaires were based either on objective sporting achievements or on self-perception, such as the MPAM-R [12,13,26,27], means that this new version collects accurate information to provide valuable information to health authorities and sports and health professionals about the variety of reasons people have for participating. This information can be applied to improve sports engagement and serve a variety of purposes, not just health-based reasons, which have traditionally been considered important reasons for physical activity.

### Limitations and Prospects

The present study has limitations in terms of the different demographic variables introduced, since, as in the study by Chowdhury DR [19], the different motivational factors will depend on the physical activity performed, sex and age, among others. Another limitation that can be associated with the study is the age range established as an inclusion criterion. Currently, life expectancy in the Spanish population and physical activity extends beyond 53 years, therefore it would be interesting to carry out studies where the inclusion criteria include subjects over 53 years, such as the study by Chowdhury DR [19] including participants aged from 18 to 71 years, and Zach S [20], who recruited participants aged from 9 to 89 years.

In terms of future lines of research, it would be interesting to deepen our knowledge of the motivational processes towards the practice of physical activity. Furthermore, it is necessary to study the relationship between the motives for exercise and other variables, such as psychological mediators (perceived competence, autonomy and relationship with others), mechanisms of regulation of exercise behavior, phases of initiation and maintenance of exercise programs, etc.), which would make it possible to establish defined motivational profiles that would enable the promotion of the practice of physical activity. Another line of research would be the in-depth study and definition of motivational profiles for sport and leisure, measuring the motivational mechanisms of the subjects to encourage them to continue practicing sport in the future and thus enhancing their sporting commitment [27,28,29,30].

## 5. Conclusions

In conclusion, the PALMS can be considered a valid and reliable instrument to measure the motivation underlying the practice of non-competitive physical activity, although further research is needed to address this area, as well as its application in different contexts in which this physical activity can be performed.

## Figures and Tables

**Table 1:** Socio-demographic data of the participants.

	*n* (%)	Mean (SD)
**Age**		35.8 (11)
**Gender**		
Male	343 (57.6)	
Female	253 (42.4)	
**Civil status**		
Single	257 (43.1)	
Partner	175 (29.4)	
Married	125 (21.0)	
Others	39 (6.5)	
**Educational level**		
Primary	27 (4.5)	
Secondary/vocational training	202 (33.9)	
University	367 (61.6)	
**Practice voluntary physical activity (hours/week)**		
1–5	200 (33.6)	
6–10	252 (42.3)	
11–15	84 (14.1)	
16–20	40 (6.7)	
>20	20 (3.4)	
**Fitness status (personal perception)**		
Good	376 (63.1)	
Regular	174 (29.2)	
Bad	46 (7.7)	
**Perceived health status**		
Good	468 (78.5)	
Regular	90 (15.1)	
Bad	38 (6.4)	

**Table 2:** Exploratory factor analysis.

		Factor							
Item	Communality	1	2	3	4	5	6	7	8
P5	0.61	0.60							
P16	0.73	0.64							
P19	0.68	0.66							
P24	0.76	0.71							
P31	0.68	0.70							
P10	0.83		0.82						
P12	0.85		0.83						
P15	0.88		0.85						
P28	0.81		0.82						
P33	0.69		0.65						
P4	0.69			0.69					
P8	0.75			0.81					
P20	0.81			0.87					
P30	0.68			0.70					
P38	0.79			0.84					
P2	0.78				0.64				
P9	0.81				0.75				
P14	0.73				0.78				
P22	0.85				0.75				
P35	0.70				0.76				
P11	0.79					0.77			
P23	0.82					0.71			
P32	0.86					0.83			
P36	0.73					0.76			
P40	0.84					0.83			
P1	0.75						0.85		
P7	0.72						0.82		
P18	0.71						0.79		
P21	0.59						0.68		
P26	0.69						0.82		
P3	0.73							0.49	
P13	0.79							0.62	
P25	0.81							0.64	
P34	0.81							0.66	
P37	0.80							0.68	
P6	0.56								0.68
P17	0.72								0.83
P27	0.64								0.72
P29	0.56								0.70
P39	0.66								0.77
Eigenvalues		15.28	3.61	2.86	2.48	1.89	1.43	1.19	1.03
% Explained variance		18.03	9.96	9.83	9.48	9.42	8.16	5.47	3.96
% Cumulative explained variance		18.03	27.99	37.82	47.30	56.72	64.88	70.35	74.31

**Table 3:** Internal consistency, convergent validity and discriminant validity.

	*α*	*ρ*c	AVE	1	2	3	4	5	6	7	8
**1. Enjoyment**	0.83	0.95	0.79	**0.89** ^†^							
**2. Mastery**	0.91	0.89	0.63	0.62 ^‡^	**0.79**						
**3. Physical condition**	0.86	0.95	0.79	0.59	0.51	**0.89**					
**4. Affiliation**	0.84	0.90	0.64	0.44	0.58	0.23	**0.80**				
**5. Psychological condition**	0.92	0.91	0.67	0.58	0.48	0.53	0.42	**0.82**			
**6. Appearance**	0.82	0.93	0.73	0.48	0.60	0.63	0.19	0.44	**0.85**		
**7. Other’s expectations**	0.85	0.90	0.61	0.26	0.33	0.28	0.52	0.20	0.19	**0.78**	
**8. Competition/Ego**	0.90	0.83	0.70	0.51	0.28	0.23	0.47	0.21	0.57	0.59	**0.83**

*α*: Cronbach’s alpha. *ρ*c: Composite reliabilities. AVE: average variance extracted. ^†^ The square root of AVE on the diagonal. ^‡^ Correlations are below the diagonal.

**Table 4:** AFC goodness-of-fit indices.

χ^2^ (d.f.)	*p*	χ^2^/d.f.	GFI	AGFI	CFI	NFI	TLI	RMSEA (90% CI)
1514.31 (712)	<0.001	2.13	0.97	0.95	0.97	0.94	0.96	0.043 (0.039–0.047)

d.f.: degrees of freedom. Goodness of Fit Index (GFI), Adjusted Goodness of Fit Index (AGFI), Comparative Fit Index (CFI), Normed Fit Index (NFI), Tucker Lewis Index (TLI) and Root Mean Square Error of Approximation (RMSEA).

**Table 5:** Model fit indices for measurement invariance by gender.

Model	χ^2^	d.f.	χ^2^/d.f.	RMSEA (90% CI)	CFI	∆RMSEA	∆CFI
Base men	1191.09	712	1.67	0.043 (0.04–0.046)	0.981		
Base women	1235.45	712	1.74	0.041 (0.038–0.044)	0.983		
Configurational invariance	2427.28	1424	1.71	0.044 (0.043–0.048)	0.979		
Metric invariance	2432.99	1456	1.67	0.045 (0.043–0.049)	0.978	0.001	0.001
Scalar invariance	2632.47	1496	1.76	0.047 (0.045–0.050)	0.978	0.002	0.000

## Data Availability

Not applicable.

## Supplementary Materials

The following supporting information can be downloaded at: https://www.mdpi.com/article/10.3390/ijerph191610064/s1.
