# Supplementary material for: Spanish Validation of the PALMS (Physical Activity and Leisure Motivation Scale)
Source: Int J Environ Res Public Health. 2022 Aug 15;19(16):10064. doi: 10.3390/ijerph191610064 (PMC9408067; doi:10.3390/ijerph191610064)
Supplement: Supplementary file 1 [file ijerph-19-10064-s001.zip › ijerph-1842708-SI.pdf]

ESCALA DE MOTIVACIÓN PARA LA PRÁCTICA DE ACTIVIDAD FISICA Y OCIO (PALMS)

Al responder a las siguientes afirmaciones, piense en los motivos que tiene para la actividad / deporte que practica.

Trate de no perder tiempo reflexionando sobre sus respuestas. Debe ser una respuesta espontánea.

No existen respuestas correctas o incorrectas. Indique cuánto se corresponden sus motivos con cada uno de los enunciados. En cada caso, 1 indica muy en desacuerdo y 5 indica muy de acuerdo. Por favor, marque el número que mejor represente su respuesta.

Realizo actividad física….	Muy en desacuerdo	En desacuerdo	Indeciso	De acuerdo	Muy de acuerdo
1-Para ganarme la vida					
2- Porque me ayuda a relajarme					
3-Porque es interesante					
4-Porque disfruto pasando tiempo con los demás					
5-Para mejorar en una actividad					
6-Porque me desenvuelvo mejor que los demás					
7-Porque me pagan por hacerlo					
8- Para hacer actividad con otras personas					
9-Para afrontar mejor el estrés					
10-Porque ayuda a mantener un cuerpo sano					
11-Para definir el musculo, lucir mejor					
12-Estar en buena forma física					
13-Porque me hace feliz					
14- Para alejarme de las presiones					
15- Para mantener la salud física					
16- Para mejorar las habilidades existentes					
17- Para ser el mejor en el grupo					
18- Para controlar alguna patología médica					
19- Para hacer mi mejor esfuerzo personal					
20- Para hacer algo en común con amigos					
21- Porque la gente me dice que lo necesito					

22- Porque actúa como un liberador de estrés					
23- Para mejorar la forma del cuerpo					
24- Obtener nuevas habilidades / actividades					
25- Porque es divertido					
26- Porque fue prescrito por médico/ fisioterapeuta					
27- Para trabajar más duro que los demás					
28- Porque me mantiene saludable					
29- Por competir con los que me rodean					
30- Para hablar con amigos haciendo ejercicio					
31- Para mantener el nivel de habilidad que tengo actualmente					
32- Para mejorar la apariencia					
33- Para mejorar la aptitud cardiovascular					
34- Porque disfruto haciendo ejercicio					
35- Para dejar de pensar en otras cosas					
36- Para adelgazar, lucir mejor					
37- Porque me lo paso bien					
38- Para estar con amigos					
39- Estar más en forma que los demás					
40- Para mantener un cuerpo esbelto y tonificado					
