# Supplementary material for: Spanish Validation of the PALMS (Physical Activity and Leisure Motivation Scale)
Source: Int J Environ Res Public Health. 2022 Aug 15;19(16):10064. doi: 10.3390/ijerph191610064 (PMC9408067; doi:10.3390/ijerph191610064)
Supplement: Supplementary file 1 [file ijerph-19-10064-s001.zip › ijerph-1842708-English vesion.pdf]

PALMS SCALE

No.	ITEM	SUBSCALE
5	To get better at an activity	Mastery
16	To improve existing skills	Mastery
19	To do my personal best	Mastery
24	To obtain new skills/activities	Mastery
31	To keep current skill level	Mastery
3	Because it is interesting	Enjoyment
13	Because it makes me happy	Enjoyment
25	Because it is fun	Enjoyment
34	Because I enjoy exercising	Enjoyment
37	Because I have a good time	Enjoyment
4	Because I enjoy spending time with others	Affiliation
8	To do activity with others	Affiliation
20	To do something in common with friends	Affiliation
30	To talk with friends exercising	Affiliation
38	To be with friends	Affiliation
6	Because I perform better than others	Competition/Ego
17	To be best in the group	Competition/Ego
27	To work harder than others	Competition/Ego
29	To compete with others around me	Competition/Ego
39	To be fitter than others	Competition/Ego
1	To earn a living	Others' expectations
7	Because I get paid to do it	Others' expectations
18	To manage medical condition	Others' expectations
21	Because people tell me I need to	Others' expectations
26	Because it was prescribed by doctor, physio	Others' expectations
10	Because it helps maintain a healthy body	Physical condition
12	Be physically fit	Physical condition
15	To maintain physical health	Physical condition
28	Because it keeps me healthy	Physical condition
33	To improve cardiovascular fitness	Physical condition
2	Because it helps me relax	Psychological condition
9	To better cope with stress	Psychological condition
14	To get away from pressures	Psychological condition
22	Because it acts as a stress release	Psychological condition
35	To take mind off other things	Psychological condition
11	To define muscle, look better	Appearance
23	To improve body shape	Appearance
32	To improve appearance	Appearance
36	To lose weight, look better	Appearance
40	To maintain trim, toned body	Appearance
